# *Npas4* regulates synaptic development in the patch/striosome compartment and early affective vocalization in neonatal mice

**DOI:** 10.1186/s13041-026-01316-z

**Published:** 2026-05-29

**Authors:** Yen-Hui Lee, Hsiao-Ying Kuo, Shih-Yun Chen, Yu-Ning Chang, Charles R. Gerfen, Fu-Chin Liu

**Affiliations:** 1 Institute of Neuroscience, National Yang Ming Chiao Tung University, Taipei, 112304 Taiwan; 2 Institute of Anatomy and Cell Biology, National Yang Ming Chiao Tung University, Taipei, 112304 Taiwan; 3 Laboratory of Systems Neuroscience, National Institute of Mental Health, Bethesda, MD 20892 USA

**Keywords:** Npas4, Synapse, Striatum, Striosome, Vocalization

## Abstract

Early vocal communication, such as isolation-induced ultrasonic vocalization (USV) in neonatal rodents, is critical for infant survival and represents a primary readout of early affective states. The patch/striosome compartment in the striatum has been proposed to serve as a limbic-motor interface integrating emotion and motivation. Developmentally, this compartment undergoes early maturation, during which activity-dependent transcription factors might enable the functional assembly of limbic-striosomal circuits to regulate infant vocal-motor behavior. *Npas4*, an activity-dependent early-response transcription factor, controls the developmental balance of excitatory and inhibitory synaptic activity. Here, we investigated how patch/ striosome compartment-expressing *Npas4* shapes early synaptic development and neonatal vocalization. We found that *Npas4* transcripts were transiently enriched in patch/striosomes at postnatal days (P) 4 and P8, before adopting a homogenous striatal distribution by P14. To investigate its biological function, we generated a patch/striosome-specific *Npas4* conditional knockout. Anatomically, *Npas4* deletion disrupted patch/striosomal synaptic balance, evidenced by a significant reduction in excitatory (Vglut1) and an increase in inhibitory (Vgat) presynaptic terminals. Behaviorally, acoustic and Markov-chain syntactical analyses of isolation-induced USVs revealed a hyper-vocal phenotype at P8, characterized by a significant increase in total USV call numbers and augmented temporal clustering of vocal bouts and sequences. Furthermore, acoustic analysis demonstrated a shift toward more complex syllable types. While the structure of global transition networks was preserved, *Npas4* knockout mice exhibited an elevated sequence entropy rate, indicating greater moment-to-moment structural variability. Our study suggests that *Npas4* shapes the development of limbic-striosomal circuits to provide top-down gating of early affective vocalization in neonates.

## Introduction

Early vocal communication is critical for infant survival and the establishment of social attachment across mammalian species [1, 2]. In rodents, neonatal pups emit isolation-induced ultrasonic vocalization (USV) when separated from the dam and their littermates [3]. These distress signals, which peak in frequency and acoustic complexity during the first two postnatal weeks, are essential for eliciting maternal search and retrieval behaviors [4]. Because isolation-induced USVs represent one of the earliest measurable forms of social communication, their analysis provides a behavioral model for investigating the ontogeny of early vocal-motor circuits and the communication deficits observed in neurodevelopmental disorders [5].

The neural circuits of innate vocalizations involve cortical and subcortical networks, including the basal ganglia. The striatum is the principal input structure of basal ganglia circuits, playing an important role in motor control, motivation, emotion, and cost-benefit conflict decision-making [6, 7]. Anatomically and neurochemically, the striatum is organized into two distinct compartments: the patch (or striosome) and the matrix [8–12]. Striosome compartments comprise early born spiny projection neurons (SPNs) that differentiate and mature earlier than SPNs in the matrix compartment [13–18]. Striosomal neurons receive preferential inputs from limbic and prefrontal cortices and project directly to dopaminergic neurons in the substantia nigra of the midbrain [9, 19–25]. Functionally, striosome compartments have been shown to be involved in reward, motivation, and cost-benefit conflict decision-making processes [6, 26–29]. Given their early maturation window and their association with reward and emotion, the developing limbic-striosomal-dopaminergic circuits are uniquely positioned to regulate early affective states and the subsequent generation of distress-related vocalizations, such as isolation-induced USVs.

Activity-dependent genetic regulation may empower the precise structural and functional assembly of these limbic-motor striatal circuits during early postnatal periods. *Npas4* (Neuronal PAS domain protein 4) is a rapidly induced transcription factor that functions as a master transcriptional regulator [30, 31]. Unlike other immediate-early genes, *Npas4* expression is specifically triggered by neuronal depolarization and calcium influx, rather than by neurotrophic factors [30–34]. Functionally, *Npas4* plays an indispensable role in homeostatic plasticity by orchestrating gene programs that regulate the balance of excitatory and inhibitory (E/I) synaptic connections onto activated neurons [31, 33]. While the influence of *Npas4* on experience-dependent plasticity in the cortex, hippocampus, and nucleus accumbens (in the context of drug addiction) is well established [30–37], its spatiotemporal regulation and specific functional role within the developing striatum, and whether it regulates the activity-dependent maturation of the patch/striosome compartment remain largely unknown.

We hypothesized that activity-dependent transcription mediated by *Npas4* within developing striosomes is involved in shaping the neural circuits that regulate early vocal communication. In the present study, we have identified *Npas4* as a crucial molecular determinant in the early maturation of the striatal patch/striosome compartment. By demonstrating that striosome-specific deletion of *Npas4* alters excitatory and inhibitory synaptic inputs to striosome SPNs, amplifies vocal output, increases acoustic complexity, and elevates sequence entropy without fundamentally rewiring innate global syntax, our study suggests that *Npas4* shapes the developing limbic-striosomal circuits to provide top-down gating of early affective vocalization in neonates. Our study highlights the importance of activity-dependent transcription in the functional assembly of limbic-striatal circuits and provides a novel framework for understanding the neurobiology of early mammalian vocal communication.

## Results

### Preferential expression of *Npas4* in developing striosome compartments of the neonatal striatum

To assess the spatiotemporal expression pattern of *Npas4* mRNA within the developing striatum, we performed double fluorescent in situ hybridization and immunohistochemistry on postnatal day (P) 4, P8 and P14 mouse brains. Brain sections were hybridized with *Npas4* riboprobes followed by immunostaining for the mu-opioid receptor 1 (MOR1), a well-established marker for striosome loci [8–10]. The results showed that *Npas4* mRNA exhibited a highly heterogeneous, patch-like distribution throughout the neonatal striatum at P4 and P8.

**Fig. 1:**
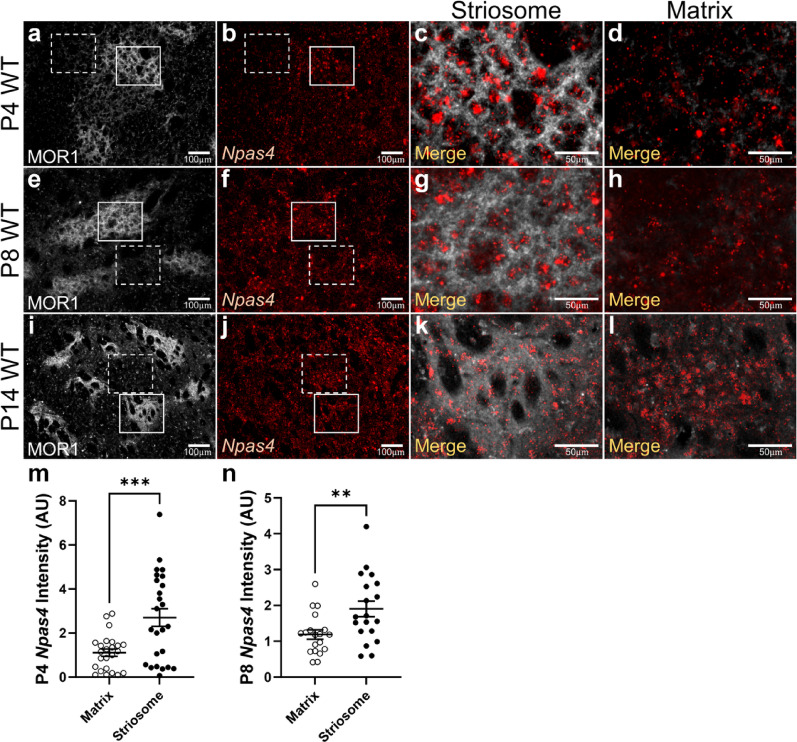
Preferential expression of *Npas4* in the developing striosome compartment of the neonatal striatum in wild type mice (**a–l**) Double fluorescent in situ hybridization for *Npas4* mRNA (red) and MOR1 immunostaining (gray, marking striosomes) at postnatal days (P) 4 (**a–d**), P8 (**e–h**), and P14 (**i–l**) striatum of wild type mice. High-magnification merges of striosomes (solid boxes shown in **c**,** g**,** k**) and matrix regions (dashed boxes shown in **d**,** h**,** l**) demonstrate *Npas4* enrichment in MOR1-positive patches at P4 and P8, transitioning to a homogeneous distribution by P14. (**m**,** n**) Quantification confirms significantly higher *Npas4* intensity in striosomes relative to the matrix at P4 (**m**) and P8 (**n**). Each dot represents one measurement (P4: matrix, 1.109 ± 0.165, *n* = 24; striosome, 2.702 ± 0.402, *n* = 25. P8: matrix, 1.188 ± 0.130, *n* = 19; striosome, 1.901 ± 0.217, *n* = 19. *N* = 2 mice/time point). Bars indicate mean ± SEM. **p < 0.01, ***p < 0.001 (Student’s *t*-test). Scale bars: 100 μm (**a**,** b**,** e**,** f**,** i**,** j**); 50 μm (**c**,** d**,** g**,** h**,** k**,** l**)

At P4, these distinct *Npas4*-positive clusters co-registered with MOR1-positive patches (Fig. 1a-d). This compartmentalized expression pattern persisted at P8, where *Npas4* enrichment continued to correspond with the MOR1-positive striosomes (Fig. 1e-h). Quantitative analysis confirmed that *Npas4* mRNA levels were significantly elevated in the striosome compartments, exhibiting marked 2.4-fold and 1.6-fold increases relative to the surrounding MOR1-negative matrix at P4 and P8, respectively (Fig. 1m, n). By P14, however, this patchy distribution transformed into a homogeneous pattern (Fig. 1i-l), indicating that *Npas4* becomes equally expressed across both the striosome and matrix compartments without regional preference. Together, these results demonstrate that *Npas4* expression is transiently and highly enriched within striosome compartments during a critical early neonatal window.

### Conditional deletion of *Npas4* in developing striosome compartments

**Fig. 2:**
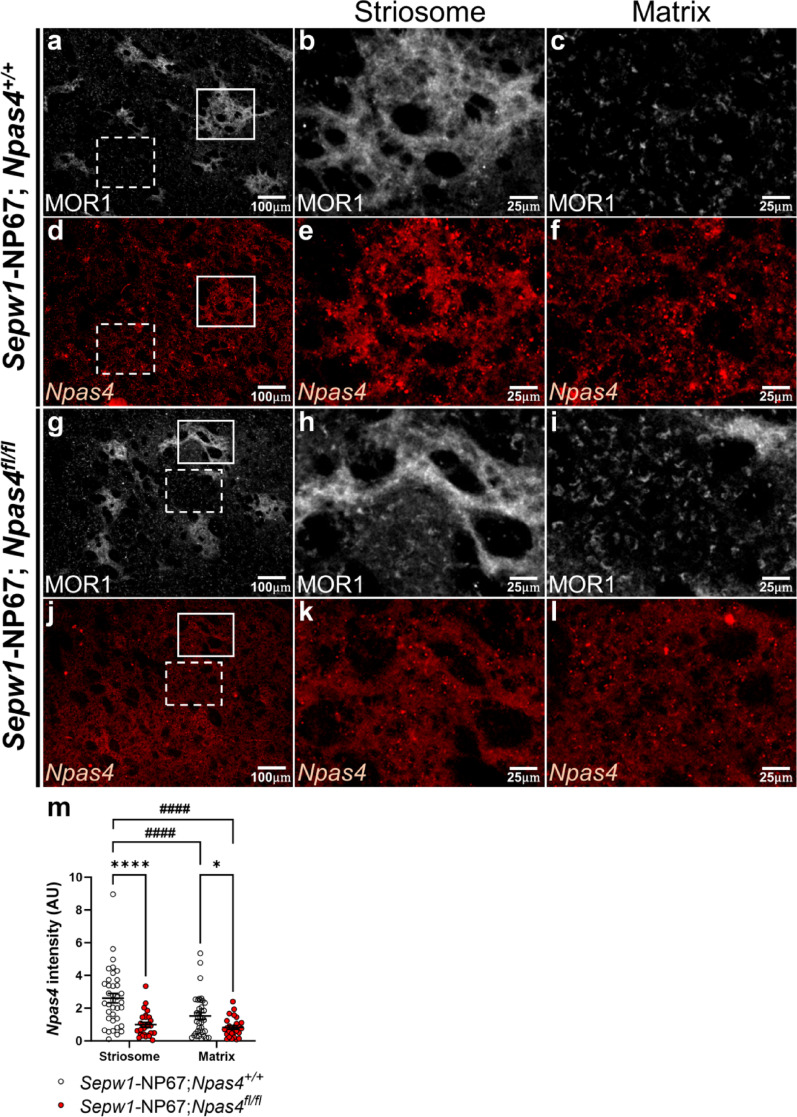
Conditional deletion of *Npas4* in developing striosome compartments (**a–l**) Double fluorescent in situ hybridization for *Npas4* mRNA (red) and MOR1 immunostaining (gray) in the striatum of P8 control (*Sepw1*-NP67;*Npas4*^+/+^) and conditional knockout (cKO; *Sepw1*-NP67;*Npas4*^fl/fl^) mice. High-magnification merges of striosomes (solid boxes in **d**,** j** shown in **e**,** k**) and matrix regions (dashed boxes in **d**,** j** shown in **f**,** l**) demonstrate marked *Npas4* reduction in cKO mice compared to controls. **(m)** Quantification of *Npas4* intensity confirms significant deletion in cKO striosomes and matrix (reflecting exo-patch cell recombination). Each dot represents one measurement (Control: striosome, 2.614 ± 0.292, *n* = 37; matrix, 1.522 ± 0.202, *n* = 38. cKO: striosome, 0.994 ± 0.147, *n* = 27; matrix, 0.822 ± 0.114, *n* = 27. *N* = 3 mice/group). Bars indicate mean ± SEM. **p < 0.01, ***p < 0.001 (Two-way ANOVA with Tukey’s post hoc test). Scale bars: 100 μm (**a**,** d**,** g**,** j**); 25 μm (**b**,** c**,** e**,** f**,** h**,** i**,** k**,** l**)

To assess the in vivo function of *Npas4*, we generated a striosome-specific conditional knockout model by intercrossing *Npas4* floxed mice [30] with the BAC-Cre line *Sepw1-*NP67 driver [38]. In *Sepw1*-NP67;*Npas4*^*fl/fl*^ conditional knockout (cKO) brains at P8, *Npas4* mRNA levels were significantly reduced by 62% within the MOR1-positive striosome compartments compared to the control *Sepw1*-NP67;*Npas4*^*+/+*^ mice (Control: striosome, 2.61 ± 0.29, *n* = 37; cKO: striosome, 0.99 ± 0.15, *p* = 0.000000163, *n* = 27; *N* = 3 mice, Fig. 2a, b, d, e, g, h, j, k, m). In P8 control brains, *Npas4* mRNA expression was notably lower in the surrounding MOR1-negative matrix regions. Despite this lower level, *Npas4* transcripts were still reduced by 46% in the matrix compartment of the *Npas4* cKO mice (Control: matrix, 1.52 ± 0.20, *n* = 38; cKO: matrix, 0.82 ± 0.11, *p* = 0.0114, *n* = 27; *N* = 3 mice, Fig. 2a, c, d, f, g, i, j, l, m). This matrix-localized reduction is likely driven by the targeted deletion of *Npas4* in “exo-patch” cells. These displaced exo-patch cells in the matrix zones are homologous to classical patch/striosome neurons and are known to express Cre recombinase activity by the *Sepw1*-NP67 driver [21].

### Conditional deletion of *Npas4* alters the development of excitatory and inhibitory presynaptic inputs

**Fig. 3:**
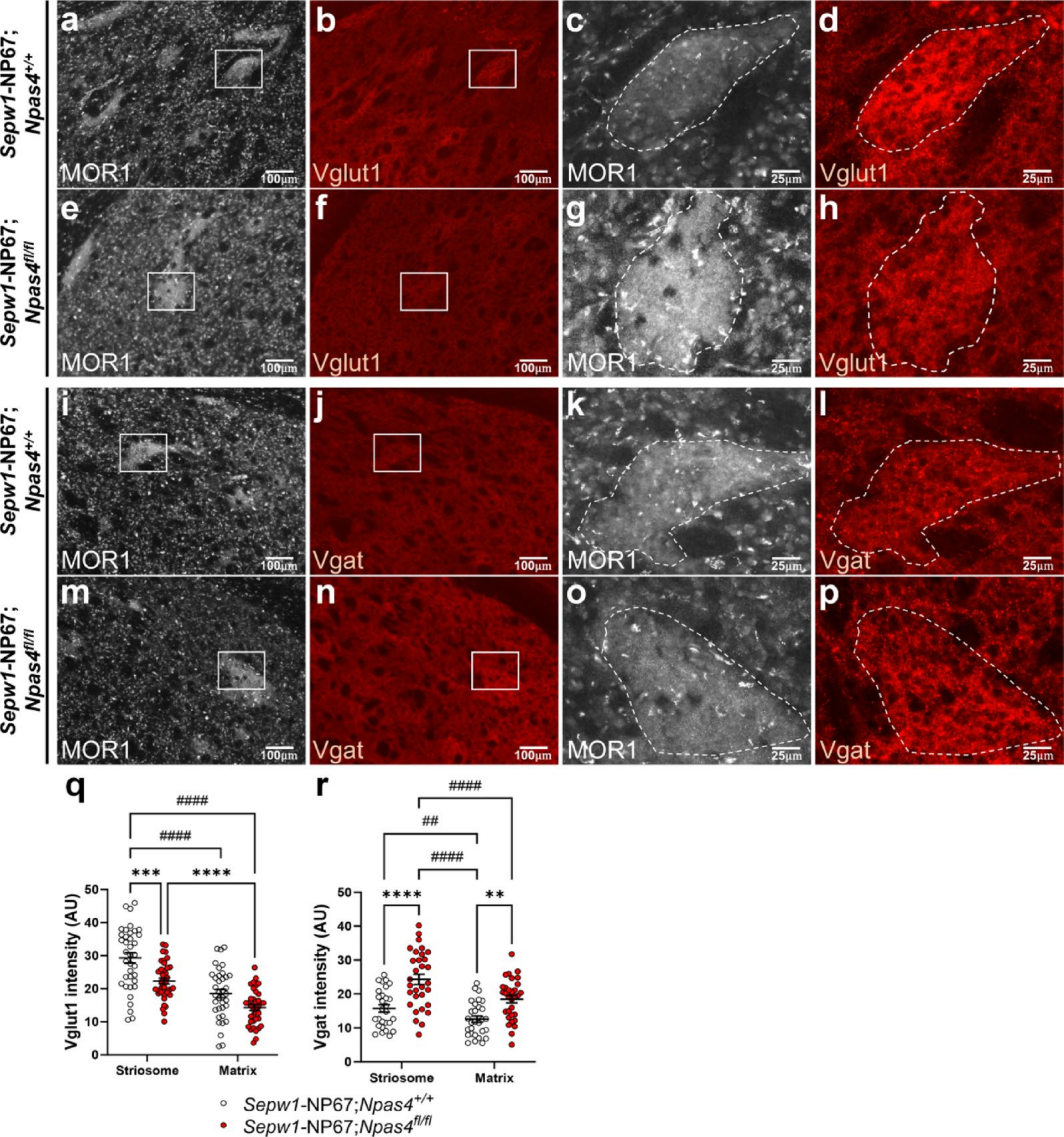
Conditional deletion of *Npas4* alters excitatory and inhibitory presynaptic inputs in the developing striatum **(a–p)** Immunostaining for presynaptic markers and MOR1 (gray) in the P8 striatum of control (*Sepw1*-NP67;*Npas4*^+/+^) and cKO (*Sepw1*-NP67;*Npas4*^fl/fl^) mice. **(a–h)** Excitatory Vglut1 (red) is reduced in cKO striosomes (**h**) versus controls (**d**; dashed lines in high-magnification views mark striosomes). **(i–p)** Inhibitory Vgat (red) is markedly increased in cKO striosomes and matrix (**p**) versus controls (**l**; dashed lines in high-magnification views mark striosomes). **(q**,** r)** Quantification confirms significantly reduced Vglut1 in cKO striosomes, and significantly increased Vgat across both compartments. Vglut1 **(q)**: Control (striosome, 29.33 ± 1.55, *n* = 37; matrix, 18.53 ± 1.25, *n* = 37); cKO (striosome, 22.31 ± 0.93, *n* = 38; matrix, 14.28 ± 0.87, *n* = 38). Vgat **(r)**: Control (striosome, 15.73 ± 1.08, *n* = 28; matrix, 12.57 ± 0.94, *n* = 29); cKO (striosome, 24.31 ± 1.51, *n* = 31; matrix, 18.44 ± 1.09, *n* = 31). *N* = 3 mice/genotype. Bars indicate mean ± SEM. **p < 0.01, ***p < 0.001, ****p < 0.0001; ^##^*p* < 0.01, ^####^*p* < 0.0001 (Two-way ANOVA with Tukey’s post hoc test). Scale bars: 100 μm (**a**,** b**,** e**,** f**,** i**,** j**,** m**,** n**); 25 μm (**c**,** d**,** g**,** h**,** k**,** l**,** o**,** p**)

To investigate the downstream cellular effects of *Npas4* deletion on striosomal circuit wiring, we assessed the distribution of synaptic inputs onto striosomal neurons. We immunostained P8 brain sections for vesicular glutamate transporter 1 (Vglut1) and vesicular GABA transporter (Vgat), which serve as markers for excitatory and inhibitory presynaptic terminals, respectively. Quantitative analysis showed that the conditional knockout of *Npas4* profoundly affected the development of these presynaptic markers in P8 striatum. Within the striosome compartments of *Sepw1-*NP67;*Npas4*^*fl/fl*^ cKO mice, the expression of the excitatory marker Vglut1 was significantly decreased by 24% compared to the control *Sepw1-*NP67;*Npas4*^*+/+*^ mice (Control: striosome, 29.33 ± 1.55, *n* = 37; cKO: striosome, 22.31 ± 0.93, *p* = 0.0003, *n* = 38; *N* = 3 mice, Fig. 3a-h and q). A downward trend was also observed in the surrounding matrix compartment, which exhibited a 23% reduction in Vglut1 (Control: matrix, 18.53 ± 1.25, *n* = 37; cKO: matrix, 14.28 ± 0.87, *n* = 38; *N* = 3 mice, Fig. 3a-h and q, *p* = 0.0658).

In dramatic contrast, inhibitory synaptic innervation was markedly enhanced in the P8 *Npas4* cKO striatum. We observed a significant 57% increase in Vgat expression specifically within the striosome compartment (Control: striosome, 15.73 ± 1.08, *n* = 28; cKO: striosome, 24.31 ± 1.51, *p* = 0.000158, *n* = 31; *N* = 3 mice, Fig. 3i-p and r). Notably, this marked upregulation of inhibitory terminals extended beyond the patch boundaries, with the matrix compartment also exhibiting a significant 47% increase in Vgat expression (Control: matrix 12.57 ± 0.94, *n* = 29; cKO: matrix, 18.44 ± 1.09, *p* = 0.0043, *n* = 31; *N* = 3 mice, Fig. 3i-p and r). As previously discussed, *Npas4* deletion also targets exo-patch cells dispersed throughout the matrix, which likely accounts for this collateral increase in local Vgat expression.

Together, these findings demonstrate that the loss of *Npas4* in developing striosomes triggers a significant reorganization of local presynaptic networks.

### Conditional deletion of *Npas4* in striosomes increases the frequency and temporal clustering of early vocalizations

**Fig. 4:**
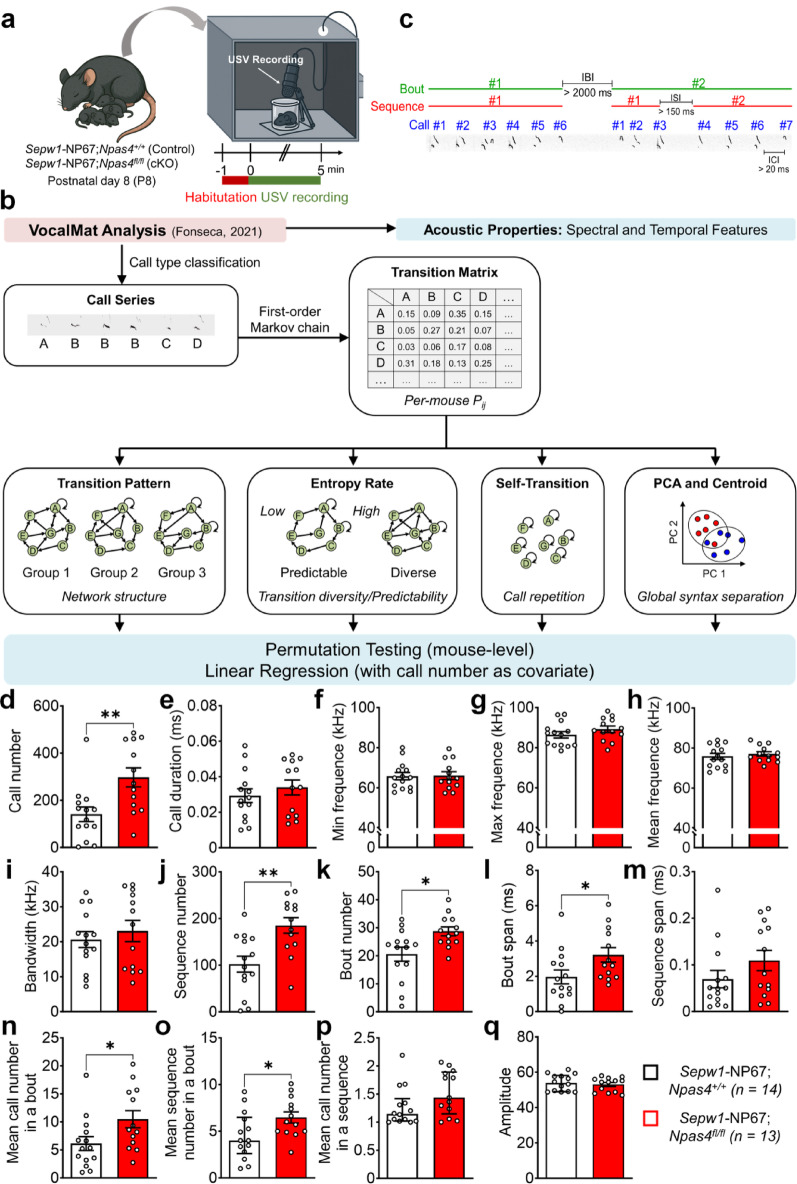
Conditional knockout of *Npas4* in the striosomal compartment increases the frequency and temporal clustering of neonatal isolation-induced vocalizations (**a**) Illustrated experimental design. (**b**) Pipeline of USV analysis using VocalMat. Annotation of USV, first-order Markov chain analysis, and permutation tests were performed with custom MATLAB scripts. (**c**) Example spectrogram showing the temporal clustering defined by inter-bout interval (IBI > 2000 ms), inter-sequence interval (ISI > 150 ms), and inter-call interval (ICI > 20 ms). (**d-q**) Quantification of acoustic USV features. Conditional *Npas4* deletion increased vocal output, as reflected by higher numbers of calls (**d**), sequences (**j**), and bouts (**k**). Mean spectral features of calls, including minimum (**f**), maximum (**g**), and mean frequency (**h**), bandwidth (**i**), and amplitude (**q**), were not significantly different between groups. Temporal organization of vocalizations was also altered, with increased calls per bout (**n**), sequences per bout (**o**), and bout span (**l**) in conditional knockout mice. *p < 0.05; **p < 0.01. Student’s *t* test in **f, g, h, j, k, l, n, o, q**. Mann-Whitney *U* test in **d, e, i, m, p**

To determine whether the activity-dependent gene *Npas4* within the striosome compartment regulates early vocal communication, we analyzed isolation-induced ultrasonic vocalizations (USVs) in neonatal mice. Vocalizations were recorded from the control mice (*n* = 14) and *Npas4* cKO mice (*n* = 13) (Fig. 4a).

USVs are not emitted randomly but are hierarchically organized into distinct temporal clusters. Individual calls (hold time more than 20 ms) are grouped into sequences (separated by inter-sequence intervals more than 150 ms), and then these sequences are further clustered into bouts (separated by longer inter-bout intervals more than 2000 ms). Analysis of the overall vocal output showed that the targeted deletion of *Npas4* significantly increased vocal output. We observed a significant increase in the total number of USV calls emitted by the *Npas4* cKO mice compared to controls (Control: 140.7 ± 30.94, *n* = 14; cKO: 297.1 ± 40.28, *p* = 0.0054, *n* = 13, Fig. 4c, d).

Beyond simply making more calls, temporal clustering of these vocalizations was broadly enhanced in *Npas4* cKO mice. *Npas4* cKO mice exhibited a highly significant increase in the total number of sequences (Control: 102.1 ± 17.07, *n* = 14; cKO: 185.1 ± 16.67, *p* = 0.0019, *n* = 13, Fig. 4j), alongside significant increases in the total bout numbers (Control: 20.57 ± 2.5, *n* = 14; cKO: 28.69 ± 1.58, *p* = 0.0123, *n* = 13, Fig. 4k) and the overall bout span (Control: 1.97 ± 0.39, *n* = 14; cKO: 3.21 ± 0.41, *p* = 0.0368, *n* = 13, Fig. 4l). Furthermore, the density of these clusters was elevated, as evidenced by significant increases in both the mean call numbers and the mean sequence numbers (Control: mean call numbers 6.13 ± 1.24, *n* = 14; cKO: mean call numbers 10.47 ± 1.54, *p* = 0.036, *n* = 13; Control: mean sequence numbers 4.50 ± 0.68, *n* = 14; cKO: mean call numbers 6.47 ± 0.59, *p* = 0.0386, *n* = 13, Fig. 4n, o). Together, these metrics indicate that the loss of striosomal *Npas4* results in a robust increase in vocal output characterized by longer and more frequent bursts of distress calls.

### Loss of striosomal *Npas4* alters the call-type composition

**Fig. 5:**
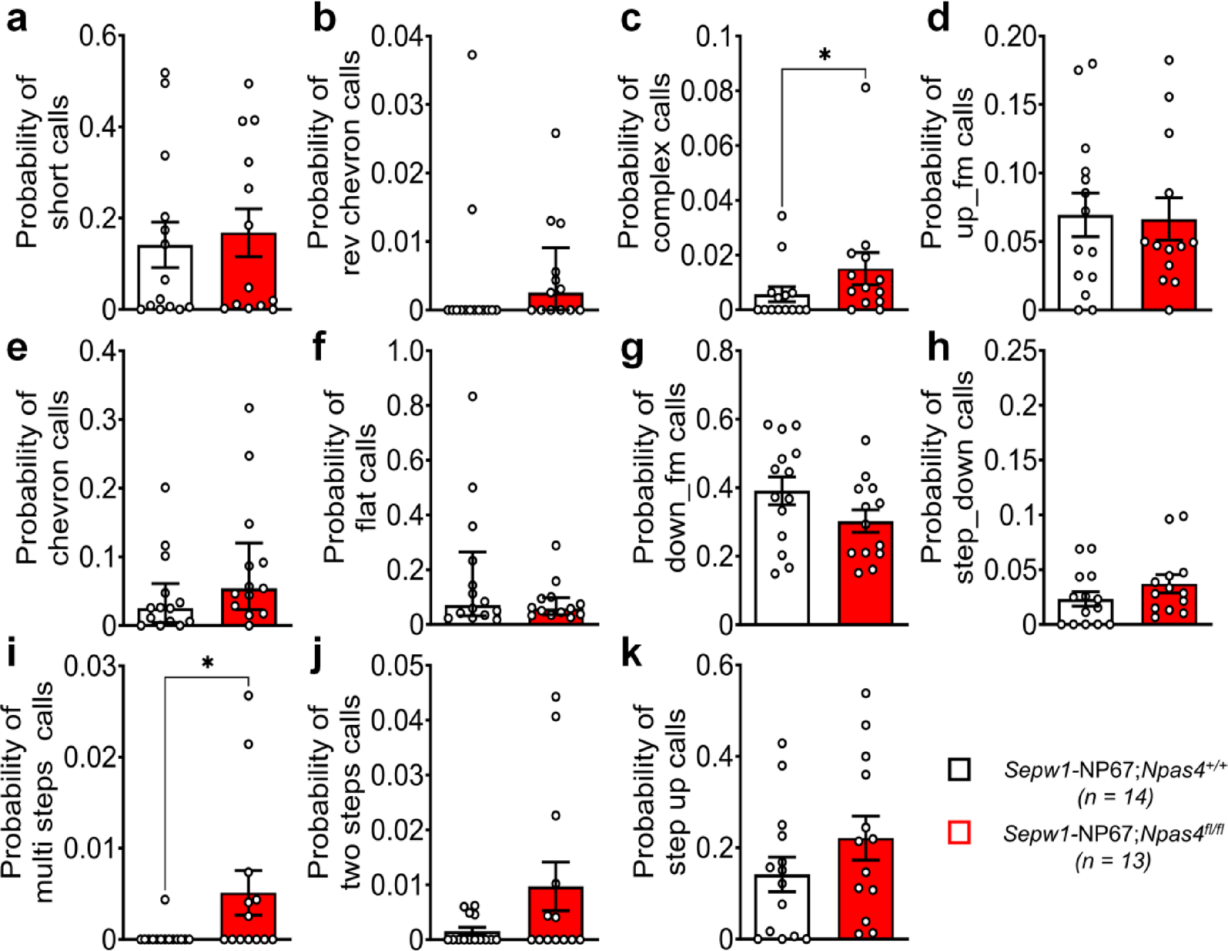
Loss of striosomal *Npas4* alters call-type composition in neonatal isolation-induced vocalizations. Calls were classified into predefined categories based on VocalMat [39], and the proportion of each call type was normalized to the total number of calls per mouse. Conditional *Npas4* knockout mice showed increased proportions of complex (**c**) and multi-step calls (**i**) compared with wildtype controls, whereas other call types were not significantly different (**a**,** b**,** d-h**,** j**,** k**). *p < 0.05. Student’s *t* test in **g, k**. Mann-Whitney *U* test in **a, b, c, d, e, f, h, i, j**

To assess whether the usage of call types changed after conditional knockout of *Npas4* in the striosomal compartment, we classified and annotated individual calls based on a previous study [39]. The call types were classified into 11 types: Short, Flat, Chevron, Reverse chevron, Down fm, Up fm, Complex, Multi steps, Two steps, Step down, and Step up using VocalMat (Fig. 4b). The *Npas4* conditional knockout mice emitted more “complex” and “multi-step” calls compared to the wildtype control mice (Control: complex 0.00568 ± 0.00276, *n* = 14; cKO: complex 0.01501 ± 0.00592, *p* = 0.0278, *n* = 13; Control: multi-step 0.00031 ± 0.00031, *n* = 14; cKO: multi-step 0.00512 ± 0.00244, *p* = 0.0213, *n* = 13, Fig. 5c, i). These results suggest an alteration in call-type composition, primarily driven by increased usage of more complex call types.

### Loss of striosomal Npas4 increases sequence variability but preserves global transition structure

Given the significant quantitative increases in call and bout frequencies and usage of call types, we next sought to investigate whether the structural sequencing (syntax) of these calls was also altered. We constructed first-order Markov chains to calculate the conditional probabilities of transitioning from one call type to another for each genotype (Fig. 4b).

**Fig. 6:**
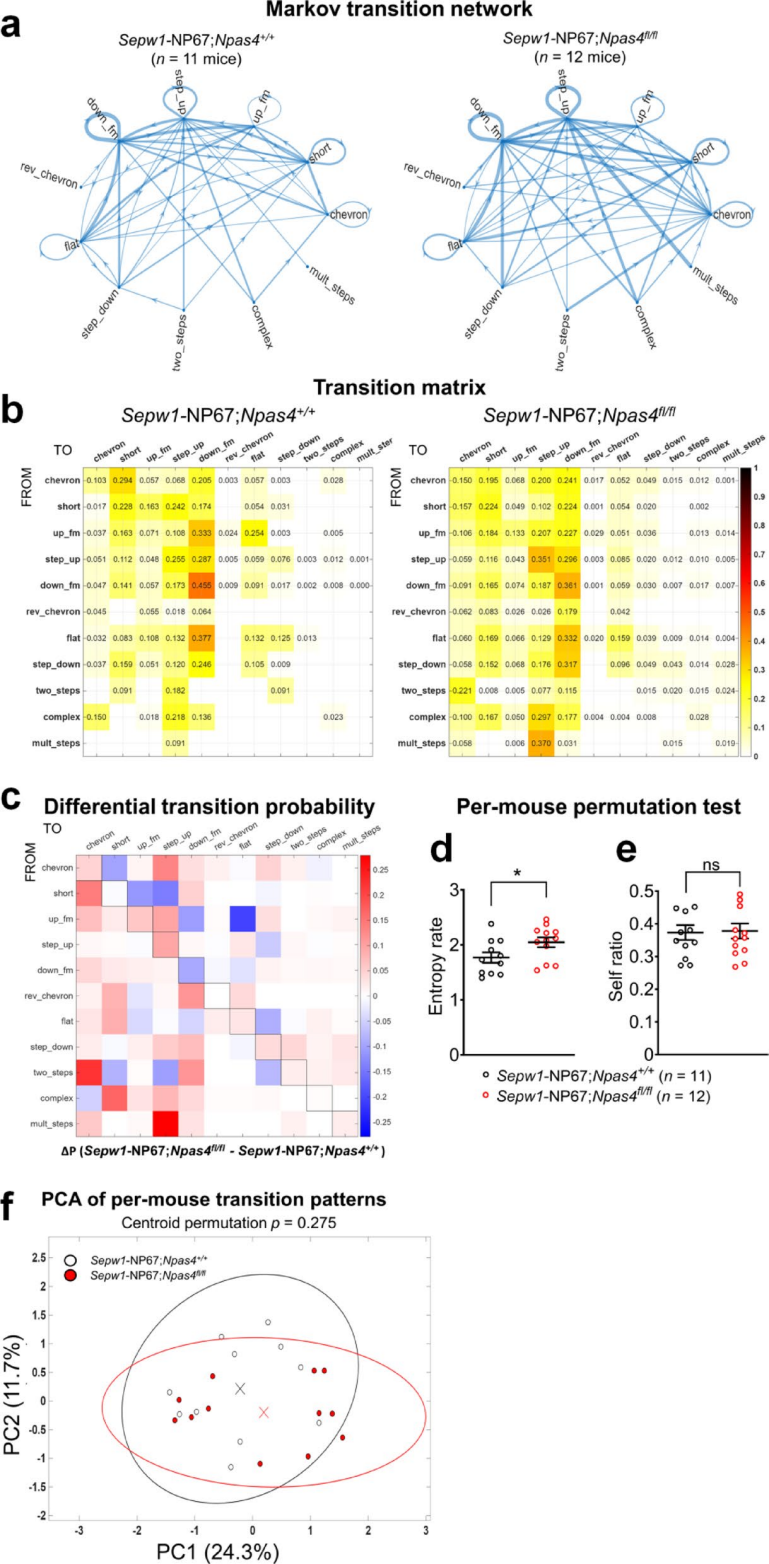
Loss of striosomal *Npas4* increases the entropy of USV transition structure without altering global transition patterns. (**a**) Markov-based transition networks depicting USV syllable sequences for each group. Nodes denote call categories, and directed edges represent conditional transition probabilities between successive calls, with edge thickness proportional to transition strength. Networks were generated from group-averaged transition matrices (threshold ≥ 0.05). (**b**) Transition probability matrices at the group level. Each element reflects the probability of transitioning from the current call type (Y-axis) to the subsequent call type (X-axis), with color intensity indicating transition likelihood. (**c**) Difference matrix of transition probabilities (ΔP; conditional knockout minus control). Rows correspond to preceding call types and columns to subsequent call types. Warmer tones indicate increased transitions, whereas cooler tones indicate reduced transitions in conditional knockout mice. Diagonal outlines denote self-transitions. (**d**) Per-animal permutation analysis of transition entropy. Conditional knockout mice showed increased entropy rates compared with controls. (**e**) Per-animal permutation analysis of self-transition probability. No group differences were detected. (**f**) Principal component analysis of individual transition profiles. Each point represents a single mouse. Ellipses indicate group dispersion, and crosses mark group centroids. Group separation was not apparent in PC space. *p < 0.05(a)(a)

At first glance, the overall networks are not obviously changed, although some transitions appeared to occur more frequently in *Npas4* cKO mice (Fig. 6a). Visualizing these transition matrices as heatmaps revealed that both wildtype control and *Npas4* cKO mice utilized a diverse repertoire of call transitions (Fig. 6b). The probabilities of transitions ending in “step_up” and “down_fm” appeared to differ between groups (Fig. 6b). However, no significant difference in the overarching transition patterns was detected (Control: *n* = 11; cKO: *n* = 12, *p* = 0.25307, Fig. 6b). A subtractive heatmap revealed an uneven pattern of alterations between transitions (Fig. 6c). To evaluate sequence predictability, we further calculated the entropy rate of the call transitions. Strikingly, *Npas4* cKO mice exhibited a modest but significantly higher entropy rate compared to wildtype controls (Control: 1.77 ± 0.10, *n* = 11; cKO: 2.05 ± 0.09, *n* = 12, *p* = 0.04446; d = 0.897, Fig. 6d). This elevation in entropy indicates that while the fundamental transition structure and global repertoire remain intact, the specific sequence of calls emitted by the *Npas4* cKO possesses greater variability, rendering their vocalizations significantly less predictable from one call to the next. Notably, neither a significant association between call number and entropy rate detected by linear regression (Control: *n* = 11; cKO: *n* = 12, *p* = 0.127), nor the alteration of self-type transition ratio (Control: *n* = 11; cKO: *n* = 12, *p* = 0.74953; Fig. 6e) was found. The subtractive heatmap revealed an uneven pattern of transition-specific differences (Fig. 6c), indicating that changes are not uniformly distributed across transitions.

To evaluate whether there was any global separation in the syntactical patterns between the two groups, we performed a principal component analysis (PCA) on the per-mouse transition patterns. The resulting PCA scatter plot demonstrated highly overlapping clusters of individual mice from both genotypes, and a centroid permutation test confirmed no significant separation in global syntax (Control: *n* = 11; cKO: *n* = 12, *p* = 0.275; Fig. 6f).

Collectively, these results demonstrate that *Npas4* in the striosomal compartment acts as a negative regulator of early vocal communication; its deletion not only amplifies USV output and temporal clustering, but also increases the structural variability of the vocal sequences, without fundamentally altering the overall transition structure.

## Discussion

In the present study, we provide the first evidence implicating the immediate early gene *Npas4* in the functional maturation of the striatal patch/striosome compartment and the regulation of early affective vocal communication. Our results demonstrate that *Npas4* mRNA is transiently enriched in neonatal striosome compartments, co-localizing with MOR1-positive patches, during a postnatal window (P4–P8), before adopting a homogeneous striatal distribution by P14. Furthermore, conditional deletion of *Npas4* within this specific compartment using the *Sepw1*-NP67 driver resulted in a hyper-vocal phenotype characterized by increased call numbers, enhanced temporal clustering, a shift toward more complex syllable types, and elevated sequence entropy. Together, these findings identify an Npas4-mediated regulation of the limbic-striatal circuits that modulate early distress vocalizations.

### Early *Npas4* expression in developing patch/striosome compartments

The basal ganglia undergo profound functional and structural reorganization during the first two postnatal weeks in rodents, a period that coincides with the peak emission of isolation-induced USVs [3, 4]. Striatal projection neurons (SPNs) receive excitatory input from the cerebral cortex and the thalamus. Corticostriatal axons arborize in the striatum as early as P2-P4 [40, 41]. *I*mmature dendritic spines of SPNs occur as early as P6, and mature spines of SPNs appear at P8-P9, followed by extensive growth of spines at P10-P28 and synaptic pruning from P18 to adulthood [42–47]. Progressive maturation of electrophysiological properties of corticostriatal circuits occurs during the first postnatal month [43, 46].

Our observation that *Npas4* expression is highly enriched in MOR1-positive striosomes at P4 and P8 aligns with the known developmental trajectory of this compartment. Striosomal SPNs are generated and differentiate early in embryogenesis and undergo circuit maturation during the early postnatal period, prior to the surrounding matrix [12, 15–18, 48–50]. By P14, the emission of isolation-induced USVs naturally declines [3, 4] and *Npas4* expression loses its striosomal preference and becomes uniformly distributed.

Because *Npas4* transcription is driven strictly by neuronal depolarization and calcium influx, it acts as a direct molecular readout of patterned network activity [30, 32]. The selective enrichment of *Npas4* in striosomes at P4 and P8 suggests that these neurons experience a surge of excitatory drive during this temporal window. This activity likely stems from the early maturation of limbic and prefrontal cortical inputs and midbrain dopaminergic inputs, which preferentially innervate the striosome compartment at neonatal stages [20, 51, 52]. Thus, the transient *Npas4* enrichment in neonatal striosome compartments we observed likely reflects a critical period of experience-dependent homeostatic plasticity, during which limbic-motor circuits are actively sculpted.

### Patch/striosome compartments as a nexus for early affective communication: limbic-patch/striosomal circuits and top-down regulation of vocal output

Our findings position the Npas4-expressing patch/striosome compartment as a critical node in the neural circuitry governing neonatal vocal communication. While the striatum is implicated in vocal regulation, our study specifically isolates the striosome compartment as a vital negative regulator of early distress vocalizations. Properly *Npas4*-mediated maturation of striosome circuits appears to exert a suppressive or filtering influence on brainstem vocal-motor output; the loss of *Npas4* disrupts this gating, resulting in disinhibited, complex, and highly variable distress signaling.

To understand how *Npas4* deletion-induced striosomal dysregulation drives this hyper-vocalization without dismantling fundamental vocal syntax, it is necessary to trace the connectivity between neonatal striatal circuits and the brainstem vocal-motor machinery. Striosome compartments serve as critical limbic-motor interfaces where emotional and reward/aversion processing converge [6, 26–29, 53, 54]. The patch/striosome compartment receives preferential projections from the limbic regions, including the prelimbic cortex, basolateral amygdala, and bed nucleus of the stria terminalis [6, 9, 19–21, 51, 52]. In turn, GABAergic striosome spiny projection neurons (SPNs) project directly to inhibit dopamine-containing neurons in the substantia nigra pars compacta (SNc), suppressing movement [9, 22–25, 54–56].

This limbic-striosomal-dopamine loop may act as an affective gating mechanism that ultimately influences the overarching sequence rules, laryngeal coordination, and respiratory timing hardwired into the lower brainstem central pattern generators (CPGs) [57–59]. However, these CPGs are fundamentally dependent on top-down permissive drive from the midbrain periaqueductal gray (PAG), the obligatory gateway for innate vocalization [60, 61]. By continuously modulating PAG activity through downstream projections, the basal ganglia may allow affective states to initiate or suppress vocal behavior.

### *Npas4* controls the excitatory-inhibitory synaptic balance in developing patch/striosome SPNs

In cortical and hippocampal circuits, *Npas4* regulates homeostatic plasticity by orchestrating gene programs that developmentally scale excitatory and inhibitory (E/I) synaptic connectivity in excitatory and inhibitory neurons [31, 33]. During the critical neonatal time window when isolation-induced USVs peak, *Npas4* is uniquely positioned at this circuit nexus to control the homeostatic scaling of limbic inputs onto striosomal SPNs. The absence of *Npas4* in striosomal neurons likely impairs their ability to properly scale synaptic inputs in response to early depolarization. Consistent with this possibility, our immunohistochemical analyses demonstrated that deletion of *Npas4* alters the synaptic architecture of the developing striatum, characterized by a significant reduction in excitatory presynaptic terminals (Vglut1) and a marked upregulation of inhibitory terminals (Vgat). In the developing striatum, the targeted loss of *Npas4* likely impairs the stabilization of descending limbic excitatory inputs, reflecting the observed drop in Vglut1. Concurrently, the robust 57% increase in Vgat suggests an aberrant proliferation of local inhibitory synapses, or a compensatory network response to developmental dysregulation. Ultimately, in the absence of *Npas4*, neonatal striosomal SPNs may fail to establish balanced synaptic inputs.

### Loss of *Npas4* drives hyper-vocalization

The behavioral hallmark of our conditional knockout model (*Sepw1-*NP67;*Npas4*^*fl/fl*^) was a marked increase in the drive to vocalize, evidenced by an elevated total call count and augmented temporal clustering (more bouts, more sequences, and increased burst density). Because isolation-induced USVs are an innate distress response driven by negative emotional states [1], it is assumed that a hyperactive limbic-striatal circuit could amplify the perceived distress or the descending motor drive to vocalize, resulting in the exaggerated, burst-like USV temporal clusters we observed.

The marked synaptic shift, a loss of excitatory drive combined with a surge in presynaptic inhibition, may contribute to the vocalization phenotypes observed in *Npas4* cKO mice. A net decrease in excitation and increase in inhibition would strongly suppress the overall firing activity of striosomal SPNs. Because these neurons normally inhibit SNc dopamine neurons, their suppression would release the SNc from its normal inhibitory constraint.

This resulting disinhibition of the SNc dopaminergic system may distort the affective gating of the basal ganglia. The overactive dopamine signal may alter descending basal ganglia signals to the PAG, providing an exaggerated, potentially erratic top-down permissive drive to the vocal CPGs. While it is not yet known whether the PAG and downstream brainstem CPGs are directly affected in *Sepw1*-NP67;*Npas4*^*fl/fl*^ mutant mice, the preservation of fundamental vocal grammar and global transition networks suggests that these core structures remain intact. Consequently, this continuous release of the brake on the vocal-motor circuitry may force the pattern generators to produce highly variable, complex calls in rapid succession, directly driving the increase in call frequency, temporal clustering, and elevated sequence entropy observed in the mutant neonates.

### Acoustic complexity and sequence entropy

A novel finding of our study is that the loss of striosomal *Npas4* not only increases the quantity of calls but also alters their acoustic composition and sequence predictability. *Npas4* cKO mice exhibited a significant increase in the use of “complex” and “multi-step” call types. In rodent models, increased syllable complexity is often correlated with heightened states of emotional arousal or distress, reflecting a more intense affective response to maternal separation [3, 4].

Intriguingly, while the mutant mice utilized a higher proportion of complex calls, the fundamental grammar of their vocalizations remained structurally intact. Principal component and overarching network analyses revealed no significant global disruption of the transition syntax, and the self-transition ratio was unchanged. However, we detected a modest but significant increase in the entropy rate of the vocal sequences, alongside specific, uneven alterations in call-type transitions. This preservation of global syntax suggests that the downstream CPGs responsible for overarching sequence rules likely remain intact [57–59, 62]. Instead, the dysregulated striosomal gating likely provides an erratic top-down drive, introducing a higher degree of variability and randomness into the vocal output. The mice select from the same innate repertoire and utilize the same broad network paths, but their moment-to-moment call sequencing becomes highly variable and less predictable.

### Limitations and future directions

It is notable that the synaptic reorganization caused by *Npas4* deletion was not strictly confined to the classic, densely packed striosome compartments; we also observed a significant 47% increase in Vgat and a near-significant 23% reduction in Vglut1 (*p* = 0.0552) within the surrounding matrix compartment. This broader phenotype can be explained by the neuroanatomical targeting of the *Sepw1*-NP67 driver. Previous studies characterizing the *Sepw1-*NP67 line have demonstrated that it labels not only the clustered SPNs within macroscopic patch/striosome compartment but also a unique subpopulation of cells known as “exo-patch” neurons [21, 38]. Exo-patch cells are dispersed throughout the matrix but are homologous to classical patch/striosome neurons. Because these matrix-embedded exo-patch cells are likely to undergo Cre-mediated *Npas4* deletion, as evidenced by the *Npas4* reduction in the matrix compartment of *Sepw1-*NP67;*Npas4*^*fl/fl*^ mice, they likely have the same cell-autonomous synaptic dysregulation as their compartmentalized counterparts. Consequently, the local presynaptic network surrounding these scattered mutant exo-patch cells undergoes similar Vglut1 reduction and Vgat upregulation, resulting in deficits across the broader striatum.

While our study establishes a link between striosomal *Npas4* and USV regulation, the specific downstream transcriptional targets of *Npas4* in developing striosome neurons remain to be identified. Future studies utilizing electrophysiology will be crucial to determine how *Npas4* deletion alters the local E/I balance of neonatal striosome SPNs. Furthermore, because early USV alterations, particularly hyper-vocalization and increased sequence entropy, are often correlated with subsequent behavioral deficits in models of neurodevelopmental disorders such as Tourette syndrome and autism spectrum disorder, in which dysfunction of the basal ganglia is implicated [47, 63], evaluating the adult socio-communicative and motor phenotypes of these *Npas4* conditional knockout mice will be valuable.

## Methods

### Animals

All animal experiments were conducted in accordance with protocols approved by the Animal Care and Use Committees of National Yang Ming Chiao Tung University. The mouse strains used in this study were the BAC-Cre line *Sepw1-*NP67 mice [38], *Npas4* floxed mice (kindly provided by Dr. Michael E. Greenberg of Harvard Medical School, USA) [30] and C57BL/6JNarl mice (National Laboratory Animal Center, Taipei, Taiwan), All animals were maintained on a C57BL/6JNarl background and housed in a specific pathogen-free environment under controlled temperature and humidity conditions, with a 12-hour light/dark cycle and ad libitum access to food and water.

## Genotyping

Genotyping was performed at postnatal day 0 (P0) by PCR using genomic DNA extracted from tail biopsies. For DNA preparation, approximately 0.2 mm of tail tissue was incubated in a solution containing 25 mM NaOH and 0.2 mM EDTA at 100 °C for 10 min. The resulting lysate was then neutralized with an equal volume of 40 mM Tris–EDTA buffer (pH 5.5) and placed on ice for cooling. PCR reactions were carried out using a thermocycler (T3000, Biometra) with the following cycling parameters: an initial denaturation at 95 °C for 5 min, followed by 31 cycles of 95 °C for 30 s, 58 °C for 30 s, and 72 °C for 30 s, with a final extension step at 72 °C for 5 min, and then held at 4 °C. For genotyping of *Sepw1-*NP67 mice and *Npas4* floxed mice, PCR was performed using the following primers: Cre316_F (5′-ATGCTTCTGTCCGTTTGCCG-3′), Cre316_R (5′ TGAGTGAACGAACCTGGTCG-3′), Npas4^fl/fl^_F (5′-CCCTGCCCTTCTAATCAGAC-3′), Npas4^fl/fl^_R (5′-GGCATTGTTCTTTCTGTCTCC-3′).

### Brain tissue preparation

The mice were placed under deep isoflurane anesthesia for a minimum of five minutes prior to tissue collection. Transcardial perfusion was subsequently performed, beginning with a 0.9% saline (NaCl) flush, immediately followed by the administration of chilled 4% paraformaldehyde (PFA) prepared in 0.1 M phosphate-buffered saline (PBS). Following removal of the brains from the skull, the brains underwent overnight post-fixation in the same 4% PFA solution at 4 °C. The tissues were then submerged in a 30% sucrose solution (in 0.1 M PBS) for a duration of at least 48 h. The brain tissues were cut into 20-µm coronal sections utilizing a cryostat (Thermo) and stored at − 70 °C.

### Synthesis of digoxigenin-labeled riboprobes

The *Npas4* riboprobes (NM_153553.5, 1639–2477 bp) were generated by PCR amplification from mouse brain cDNA using the primers NPAS4-F (5′-CCAGAAGCTTTGAAGACCAGTT-3′) and NPAS4-R (5′-CTTCGTAGGGGAATGTTGAGAC-3′) and subsequently subcloned into the pGEM-T Easy plasmid (A1360, Promega). Digoxigenin (DIG)-labeled RNA probes were generated via in vitro transcription utilizing specific RNA labeling mixes. Briefly, 1 µg of the PCR-amplified template with M13-F (5′-GTAAAACGACGGCCAGT-3′) and M13-R (5′-AACAGCTATGACCATG-3′) primers was combined with 4 µl of 5X transcription buffer, 2 µl of 0.1 M DTT, 2 µl of DIG labeling mix (11277073910, Merck), 1 µl of RNasin (N2111, Promega), and 1.5 µl of T7 RNA polymerase (P2075, Promega). The reaction was brought to a final volume of 20 µl with DEPC-treated water and incubated for 2 h at 37 °C. Following transcription, the template DNA was degraded using DNase RQ1 (M6101, Promega) at 37 °C for 30 min. Reactions were terminated by the addition of 5 µl of 0.2 M EDTA (pH 8.0) and a subsequent 5-minute incubation on ice. The labeled RNA was then mixed with 30 µl of STE buffer (10 mM Tris-HCl, pH 8.0; 1 mM EDTA, pH 8.0; 0.1 M NaCl) and 3 µl of 1 M DTT, followed by purification utilizing G-50 Mini Quick Spin Columns (11 814427001, Merk). Probe integrity was verified by assessing samples collected prior to DNase treatment alongside the post-purification products. To preserve their efficacy, the purified riboprobes were divided into smaller aliquots and maintained at − 70 °C until experimental use.

### In situ hybridization

 In situ hybridization was performed as previously described [64]. Tissue sections were air-dried at room temperature for 10 min and subsequently placed in a desiccator under vacuum for a minimum of one hour to eliminate residual moisture. Pre-treatment protocols varied by developmental stage: embryonic sections were rinsed in 1X PBS for 5 min and defatted in 0.1% Triton X-100/1X PBS for 5 min, whereas postnatal (P0) to adult sections were post-fixed in 4% paraformaldehyde (PFA) in 1X PBS on ice for 30 min prior to permeabilization with 0.3% Triton X-100/1X PBS for 15 min. Following a 1X PBS wash, all slides were submerged in 0.2 N HCl (in DEPC water) for 20 min. Crucially, a proteinase K digestion (10 µg/ml in 1X PBS, MDBio, Inc.) was performed at 37 °C for 2 to 5 min to facilitate protein removal, depending on the tissue stage. Sections were washed, post-fixed briefly in 4% PFA/1X PBS for 5 min and quenched with two 15-minute incubations in glycine (2 µg/ml in 1X PBS). Prehybridization was conducted in a humidified chamber at 65 °C for 90 min using a solution of 50% deionized formamide (Sigma) in 2X standard saline citrate (SSC; 30 mM sodium citrate, 300 mM NaCl, pH 7.0). Riboprobes were diluted (1:250 to 1:1000) in hybridization buffer (10% dextran sulfate, 50% formamide, 1 mM EDTA pH 8.0, 0.01 M Tris pH 8.0, 0.3 M NaCl, 1X Denhardt’s solution, 500 µg/ml yeast tRNA, and 10 mM DTT) and denatured at 90 °C for 10 min. Each slide received 200 µl of the probe mixture, was coverslipped, and sealed with rubber cement. Hybridization proceeded for 16 h at 65 °C. Post-hybridization washes included 5 min in 5X SSC, 1 h in 50% formamide/2X SSC at 65 °C, and a brief equilibration in buffer (10 mM Tris-HCl pH 8.0, 500 mM NaCl) before and after RNase A digestion (20 µg/ml, 37 °C for 30 min). Stringency washes consisted of two 20-minute incubations each in 2X SSC and 0.2X SSC at 65 °C. Sections were then equilibrated in TNT buffer (100 mM Tris pH 7.5, 150 mM NaCl) and blocked for 60 min with 2% blocking reagent and 20% sheep serum in TNT buffer. For fluorescent detection, 0.1% Tween-20 was added to all TNT buffers. Endogenous peroxidases were quenched with 0.1% H2O2 in TNT prior to the 60-minute blocking step. Sections were incubated overnight with a horseradish peroxidase (HRP)-conjugated sheep anti-DIG antibody (1:100, 11207733910, Roche, Basel, Switzerland). Signal amplification was achieved using the Tyramide Signal Amplification (TSA) system (PerkinElmer), applying tyramide-Cy3 (1:1000 in 1X dilution buffer, Vector) for 10 min. Slides were washed in TNT, cover slipped, and imaged utilizing a fluorescence microscope (BX53, Olympus).

### Immunofluorescence imaging and quantitative analysis

Fluorescent images were acquired using an upright microscope (BX-53 M, Olympus, Japan) equipped with a 40× objective lens. To ensure comparability across experimental groups, all images were acquired using identical exposure times and imaging parameters. Striosome compartments were identified based on µ-opioid receptor (MOR-1) immunoreactivity. Regions of interest (ROIs) corresponding to MOR-1-positive striosomes were manually delineated using Fiji (ImageJ, NIH, USA), while adjacent MOR-1-negative areas were defined as matrix compartments. Following the ROI definition, fluorescence intensities of Vglut1 and Vgat signals were quantified in both striosome and matrix regions. For each brain, background fluorescence was determined from signal-free regions within the striatum. The mean background intensity from each section was then subtracted from all fluorescence measurements obtained from sections of the same brain. All imaging acquisition and analysis parameters were kept constant across experimental groups.

*Npas4* mRNA puncta were quantified using the SynBot software [65]. Image processing was performed using consistent thresholding and segmentation settings across all samples. Puncta with an area smaller than 15 pixels were excluded from further analysis. To reduce background interference, only the top 10% highest-intensity puncta within each channel were retained. For Figs. 1 and 2, the analysis focused on *Npas4* (red) puncta. Using the output generated by the software, the mean *Npas4* fluorescence intensity was calculated for both striosome and matrix compartments. Background fluorescence, measured from signal-free regions, was subtracted from all values prior to analysis. All quantified data were compiled and used for subsequent statistical analysis.

### Ultrasonic vocalization recording and analysis

Ultrasonic vocalizations (USVs) were collected from pups at P8. After a 15–30 min habituation period in a sound-attenuating room, each pup was separated from its mother and siblings. The pup was then placed in a 6 cm diameter glass beaker inside a soundproof chamber. For recording, a CM16 condenser ultrasound microphone (Avisoft-Bioacoustics) was positioned 6 cm above the subject. Each session lasted 5 min and was captured using an Avisoft UltraSoundGate 116 and Avisoft-RECORDER software. To evaluate USV characteristics, including call types and transitions, the data were first processed with VocalMat [39]. Subsequently, we used MATLAB to perform first-order Markov chain analysis on the classification results, following established protocols [66, 67].

### USV syntax Markov-chain analysis and statistics

To ensure reliable estimation of call sequence statistics, we restricted the analysis to mice producing at least 50 USV calls. Entropy estimates are known to be biased under sparse sampling conditions [68]. Similar sequence-based analyses of mouse USVs rely on sufficient numbers of calls per animal [69]. This threshold was selected to balance estimation reliability with statistical power, as more stringent thresholds would substantially reduce sample size and increase the risk of Type II errors. The organization of USVs was analyzed using a bout-based Markov chain model, where transitions were restricted to within-bout sequences. For every mouse, individual calls were categorized into specific syllable types (states). For each subject, a state-to-state count matrix (C) was generated by totaling consecutive call transitions. Finally, the transition probability matrix (P) was derived by row-normalizing C to obtain the conditional probabilities.

$$\:{P}_{ij}=P({X}_{t+1}=j|{X}_{t}=i)$$

We derived summary metrics for each mouse based on the transition count matrix. The self-transition ratio was calculated as the sum of transitions along the diagonal of C (representing transitions to the same state) divided by the total number of transitions. Furthermore, the entropy rate (measured in bits) was determined from the transition probability matrix. This calculation was based on Shannon entropy and the empirical stationary distribution, where π_i_ represents the stationary distribution.

$$\:H=-\sum\:_{i}{\pi\:}_{i}\sum\:_{j}{P}_{ij}{log}{P}_{ij}$$

To summarize the transition structure for each experimental group, we calculated the average of the individual transition probability matrices (*P*^*(1)*^, *P*^*(2)*^, …). This process yielded a group-mean transition probability matrix (P̄) for each condition.

$$\:\stackrel{-}{P}=\frac{1}{n}\sum\:_{k=1}^{n}{P}^{\left(k\right)}$$

We used heatmaps to visualize the transition patterns within each group. These group-mean matrices were then transformed into directed syntax networks.

### Permutation tests and multiple-comparison correction

To evaluate group differences in transition structure, we performed seeded, per-mouse permutation tests with 50,000 iterations. To measure global variations in transition patterns, we used the squared Euclidean distance between the group-average transition matrices as the test statistic:

$$\:{T}_{obs}=\sum\:_{i,j}{\left({\stackrel{-}{P}}_{A,ij}-{\stackrel{-}{P}}_{B,ij}\right)}^{2}$$

To evaluate scalar metrics, specifically the entropy rate and self-transition ratio, the absolute difference between group means was used as the test statistic.

For the permutation test:

$$\:{T}_{perm}=\sum\:{\left({\stackrel{-}{P}}_{A,ij}^{perm}-{\stackrel{-}{P}}_{B,ij}^{perm}\right)}^{2}$$

Empirical P values were computed using a + 1 correction:

$$\:P=\frac{1+\#\left\{{T}_{perm}\ge\:{T}_{obs}\right\}}{{n}_{perm}+1}$$

### Linear model control

To account for the potential influence of call frequency, we performed a linear model analysis. In this model, entropy rate was treated as the response variable, while the experimental group and total call count were included as predictors. EntropyRate_i_ = β_0_ + β_1_(TotalCalls_i_) + β_2_(Group_i_) + ϵ_i_.

### PCA of transition patterns and centroid permutation test

For analysis in a reduced-dimensional space, transition probability matrices from each mouse were converted into vectors. To stabilize variance, these data underwent a square-root transformation followed by mean-centering prior to Principal Component Analysis (PCA). We conducted PCA across the entire dataset and recorded the resulting eigenvalues, as well as the individual and cumulative explained variance. To determine if groups were distinct within the PC space, we performed a centroid-based permutation test. Specifically, group labels were shuffled 50,000 times, and group centroids were recalculated in the PC1–PC2 coordinate system. The maximum distance between any two centroids served as the test statistic. All computational analyses were carried out using custom MATLAB scripts.

### Statistical analysis

All data were tested for normality using the Shapiro–Wilk test before proceeding with further statistical comparisons. For datasets showing slight deviation from normality, square root transformation was applied when necessary to meet the assumption of normality. For normally distributed datasets, independent *t*-test or two-way ANOVA followed by Tukey’s HSD post hoc test was used, and data were presented as mean ± s.e.m. For the datasets without normal distributions, the Mann-Whitney U test was used for analysis, and data were presented as median ± interquartile range. Statistical analysis was performed with SPSS (IBM, version 21).

## Data Availability

No datasets were generated or analysed during the current study.

## Abbreviations

CPGs: Central pattern generators
E/I: Excitatory and inhibitory
MOR1: mu-opioid receptor
Npas4: Neuronal PAS domain protein 4
PAG: Periaqueductal gray
PCA: Principal component analysis
SPN: Spiny projection neurons
SNc: Substantia nigra pars compacta
USV: Ultrasonic vocalization
Vgat: Vesicular GABA transporter
Vglut1: Vesicular glutamate transporters 1
